# Dichloridobis(2-phenyl­pyridine-κ*N*)zinc(II)

**DOI:** 10.1107/S1600536812010616

**Published:** 2012-03-17

**Authors:** Sivanesan Dharmalingam, Ha-Jin Lee, Sungho Yoon

**Affiliations:** a Department of Bio & Nano Chemistry, College of Natural Sciences, Kookmin University, 861-1 Jeongneung-dong, Seongbuk-gu, Seoul 136-702, Republic of Korea; b Jeonju Center, Korea Basic Science Institute (KBSI), 664-14 Dukjin dong 1-ga, Dukjin-gu, Jeonju 561-756, Republic of Korea

## Abstract

In the title compound, [ZnCl_2_(C_11_H_9_N)_2_], the Zn^2+^ cation lies on a twofold axis and is coordinated by two Cl^−^ anions and the N atoms of two 2-phenyl­pyridine ligands, forming a ZnN_2_Cl_2_ polyhedron with a slightly distorted tetra­hedral coordination geometry. The dihedral angle between the phenyl ring and the metal-bound pyridine ring is 50.3 (4)° for each 2-phenyl­pyridine ligand. This arranges the phenyl ring from one ligand in the complex above the pyridine ring of the other resulting in an intra­molecular π–π inter­action, with a centroid–centroid distance of 3.6796 (17) Å. Weak C—H⋯Cl hydrogen bonds stabilize the crystal packing, linking mol­ecules into chains along the *c* axis.

## Related literature

For background to metal complexes with 2-phenyl­pyridine ligands, see: Samha *et al.* (1993 ▶); Yoshinari *et al.* (2010 ▶); Zhao *et al.* (2008 ▶). For those involving substituted 2-phenyl­pyridine ligands, see: Santoro *et al.* (2011 ▶).
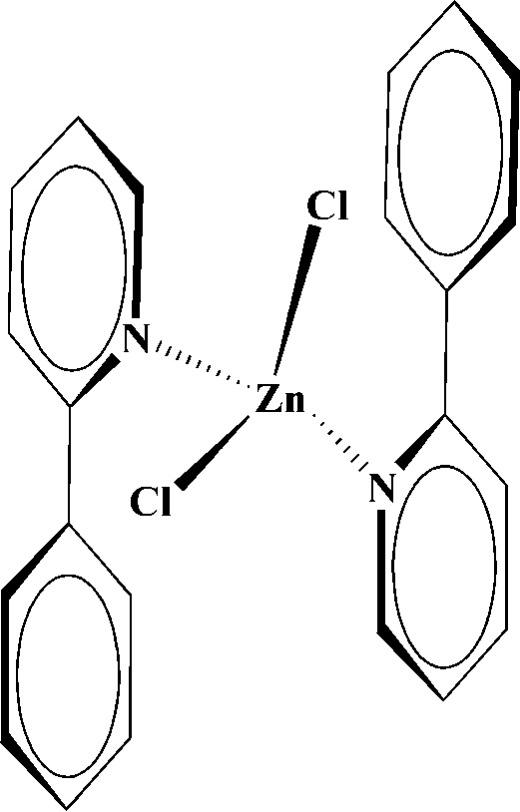


## Experimental

### 

#### Crystal data

[ZnCl_2_(C_11_H_9_N)_2_]
*M* *_r_* = 446.67
Tetragonal, 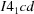

*a* = 15.2803 (3) Å
*c* = 16.4339 (7) Å
*V* = 3837.1 (2) Å^3^
*Z* = 8
Mo *K*α radiation
μ = 1.57 mm^−1^
*T* = 200 K
0.31 × 0.29 × 0.14 mm

#### Data collection

Bruker SMART CCD area-detector diffractometer
Absorption correction: multi-scan (*SADABS*; Bruker, 2000 ▶) *T*_min_ = 0.814, *T*_max_ = 1.00
13165 measured reflections
2231 independent reflections
1754 reflections with *I* > 2σ(*I*)
*R*_int_ = 0.047

#### Refinement

*R*[*F* ^2^ > 2σ(*F* ^2^)] = 0.031
*wR*(*F* ^2^) = 0.074
*S* = 1.09
2231 reflections
123 parameters
1 restraint
H-atom parameters constrained
Δρ_max_ = 0.37 e Å^−3^
Δρ_min_ = −0.36 e Å^−3^
Absolute structure: Flack (1983 ▶), 997 Friedel pairs
Flack parameter: 0.02 (2)

### 

Data collection: *SMART* (Bruker, 2000 ▶); cell refinement: *SAINT* (Bruker, 2000 ▶); data reduction: *SAINT*; program(s) used to solve structure: *SHELXS97* (Sheldrick, 2008 ▶); program(s) used to refine structure: *SHELXL97* (Sheldrick, 2008 ▶); molecular graphics: *SHELXTL* (Sheldrick, 2008 ▶); software used to prepare material for publication: *SHELXTL*.

## Supplementary Material

Crystal structure: contains datablock(s) I, global. DOI: 10.1107/S1600536812010616/sj5207sup1.cif

Structure factors: contains datablock(s) I. DOI: 10.1107/S1600536812010616/sj5207Isup2.hkl

Additional supplementary materials:  crystallographic information; 3D view; checkCIF report

## Figures and Tables

**Table 1:** Selected bond lengths (Å)

Zn1—N1	2.097 (2)
Zn1—Cl1	2.2432 (11)

**Table 2:** Hydrogen-bond geometry (Å, °)

*D*—H⋯*A*	*D*—H	H⋯*A*	*D*⋯*A*	*D*—H⋯*A*
C11—H11⋯Cl1^i^	0.95	2.90	3.666 (3)	138

Symmetry code: (i) 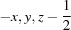.

**Fig. 1.**
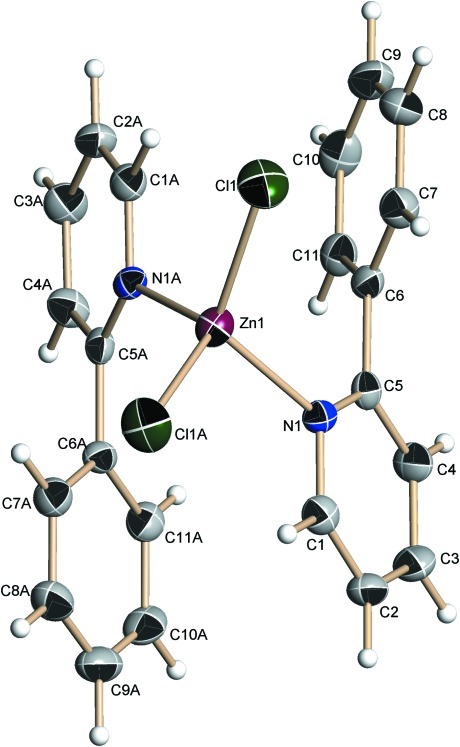
The molecular structure of the title compound, showing the atom-numbering and with displacement ellipsoids drawn at the 50% probability level.

**Fig. 2.**
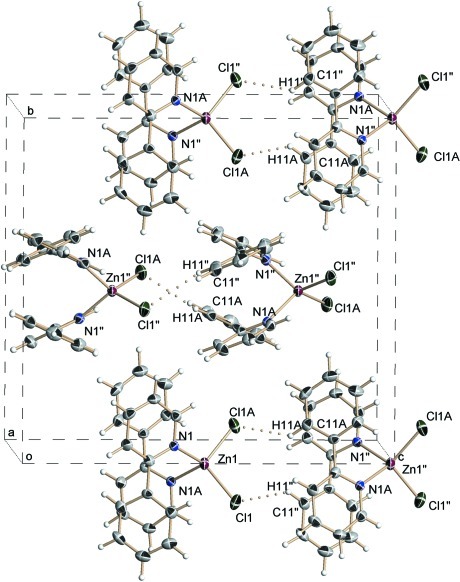
A crystal packing diagram of the title compound, viewed along the a axis.

## supplementary crystallographic information

### Comment

2-Phenylpyridine coordinated metal complexes of Ir^III^ and Pt^II^ are well known for their intense photoluminescence (Samha *et al.*, 1993; Yoshinari *et al.*, 2010; Zhao *et al.*, 2008). A four coordinate Pt^II^ square planar metal complex has also been reported with two 2-phenylpyridine and two Cl^-^ ligands (Yoshinari *et al.* 2010). Complexes with substituted 2-phenylpyridine ligands have also been reported (Santoro *et al.*, 2011). Here, we report the structure of a tetrahedrally coordinated Zn^2+^ complex which crystallizes in the tetragonal space group *I*4_1_*cd* with one half molecule in the asymmetric unit. Bond distances to the metal are given in Table 1 with the structure of the molecule shown in Fig 1 and its crystal packing involving weak intermolecular C—H···Cl interactions detailed in Fig 2 and Table 2.

### Experimental

To a solution of 2-phenylpyridine (1.56 ml, 11.0 mmol) in 30 mL of acetonitrile, ZnCl_2_ (0.50 g, 3.6 mmol) was added at room temperature. After three hours, acetonitrile was removed under reduced pressure and crystals were collected from a dichloromethane and pentane layering system. Colorless block-like crystals. Yield = 90%, (1.45 g).

### Refinement

The H atoms were placed at calculated positions and refined as riding with C–H = 0.95 Å [*U*iso(H) = 1.2 *U*eq(C)].

### Crystal data

**Table:**

[ZnCl_2_(C_11_H_9_N)_2_]	*D*_x_ = 1.546 Mg m^−^^3^
*M**_r_* = 446.67	Mo *K*α radiation, λ = 0.71073 Å
Tetragonal, *I*4_1_*c**d*	Cell parameters from 4803 reflections
Hall symbol: I 4bw -2c	θ = 2.7–28.1°
*a* = 15.2803 (3) Å	µ = 1.57 mm^−^^1^
*c* = 16.4339 (7) Å	*T* = 200 K
*V* = 3837.1 (2) Å^3^	Block, colorless
*Z* = 8	0.31 × 0.29 × 0.14 mm
*F*(000) = 1824	

### Data collection

**Table:**

Bruker SMART CCD area-detector diffractometer	2231 independent reflections
Radiation source: fine-focus sealed tube	1754 reflections with *I* > 2σ(*I*)
Graphite monochromator	*R*_int_ = 0.047
φ and ω scans	θ_max_ = 28.3°, θ_min_ = 2.7°
Absorption correction: multi-scan (*SADABS*; Bruker, 2000)	*h* = −18→20
*T*_min_ = 0.814, *T*_max_ = 1.00	*k* = −20→20
13165 measured reflections	*l* = −21→16

### Refinement

**Table:**

Refinement on *F*^2^	Secondary atom site location: difference Fourier map
Least-squares matrix: full	Hydrogen site location: inferred from neighbouring sites
*R*[*F*^2^ > 2σ(*F*^2^)] = 0.031	H-atom parameters constrained
*wR*(*F*^2^) = 0.074	*w* = 1/[σ^2^(*F*_o_^2^) + (0.0275*P*)^2^ + 1.0418*P*] where *P* = (*F*_o_^2^ + 2*F*_c_^2^)/3
*S* = 1.09	(Δ/σ)_max_ < 0.001
2231 reflections	Δρ_max_ = 0.37 e Å^−^^3^
123 parameters	Δρ_min_ = −0.36 e Å^−^^3^
1 restraint	Absolute structure: Flack (1983), 997 Friedel pairs
Primary atom site location: structure-invariant direct methods	Flack parameter: 0.02 (2)

### Special details

**Table:**

Geometry. All e.s.d.'s (except the e.s.d. in the dihedral angle between two l.s. planes) are estimated using the full covariance matrix. The cell e.s.d.'s are taken into account individually in the estimation of e.s.d.'s in distances, angles and torsion angles; correlations between e.s.d.'s in cell parameters are only used when they are defined by crystal symmetry. An approximate (isotropic) treatment of cell e.s.d.'s is used for estimating e.s.d.'s involving l.s. planes.
Refinement. Refinement of *F*^2^ against ALL reflections. The weighted *R*-factor *wR* and goodness of fit *S* are based on *F*^2^, conventional *R*-factors *R* are based on *F*, with *F* set to zero for negative *F*^2^. The threshold expression of *F*^2^ > σ(*F*^2^) is used only for calculating *R*-factors(gt) *etc*. and is not relevant to the choice of reflections for refinement. *R*-factors based on *F*^2^ are statistically about twice as large as those based on *F*, and *R*- factors based on ALL data will be even larger.

### Fractional atomic coordinates and isotropic or equivalent isotropic displacement parameters (Å^2^)

**Table:**

	*x*	*y*	*z*	*U*_iso_*/*U*_eq_	
Zn1	0.0000	0.0000	0.48912 (7)	0.02540 (12)	
N1	0.08480 (15)	−0.05713 (15)	0.40397 (14)	0.0207 (5)	
C1	0.08984 (19)	−0.14499 (19)	0.40824 (18)	0.0265 (6)	
H1	0.0670	−0.1733	0.4551	0.032*	
C2	0.12652 (19)	−0.19583 (19)	0.34786 (19)	0.0298 (7)	
H2	0.1299	−0.2576	0.3536	0.036*	
C3	0.1581 (2)	−0.1554 (2)	0.27923 (19)	0.0329 (7)	
H3	0.1834	−0.1888	0.2365	0.039*	
C4	0.1525 (2)	−0.0650 (2)	0.27340 (18)	0.0291 (7)	
H4	0.1735	−0.0361	0.2261	0.035*	
C5	0.11645 (16)	−0.01706 (17)	0.33602 (17)	0.0222 (6)	
C6	0.11275 (18)	0.07988 (17)	0.33127 (17)	0.0226 (6)	
C7	0.1446 (2)	0.13120 (19)	0.39417 (19)	0.0298 (7)	
H7	0.1688	0.1040	0.4411	0.036*	
C8	0.1416 (2)	0.2216 (2)	0.3894 (2)	0.0373 (8)	
H8	0.1634	0.2564	0.4328	0.045*	
C9	0.1067 (2)	0.2607 (2)	0.3209 (2)	0.0389 (8)	
H9	0.1040	0.3227	0.3174	0.047*	
C10	0.0756 (2)	0.2102 (2)	0.2572 (2)	0.0353 (8)	
H10	0.0520	0.2376	0.2102	0.042*	
C11	0.0787 (2)	0.1198 (2)	0.26203 (17)	0.0285 (7)	
H11	0.0577	0.0851	0.2182	0.034*	
Cl1	0.04582 (6)	0.10755 (6)	0.57169 (5)	0.0421 (2)	

### Atomic displacement parameters (Å^2^)

**Table:**

	*U*^11^	*U*^22^	*U*^33^	*U*^12^	*U*^13^	*U*^23^
Zn1	0.0278 (3)	0.0310 (3)	0.0174 (2)	0.0033 (2)	0.000	0.000
N1	0.0218 (12)	0.0206 (12)	0.0196 (12)	0.0022 (10)	−0.0029 (10)	0.0004 (10)
C1	0.0260 (17)	0.0283 (16)	0.0253 (15)	0.0001 (13)	−0.0036 (13)	0.0026 (13)
C2	0.0321 (17)	0.0206 (15)	0.0368 (18)	0.0012 (12)	−0.0012 (13)	−0.0022 (13)
C3	0.0350 (18)	0.0287 (18)	0.0350 (18)	0.0024 (14)	0.0066 (15)	−0.0096 (15)
C4	0.0316 (17)	0.0297 (17)	0.0260 (15)	−0.0030 (13)	0.0089 (13)	0.0005 (13)
C5	0.0164 (13)	0.0272 (15)	0.0229 (16)	−0.0006 (11)	−0.0030 (11)	0.0014 (12)
C6	0.0216 (14)	0.0202 (13)	0.0262 (16)	0.0004 (12)	0.0049 (12)	0.0025 (12)
C7	0.0288 (17)	0.0308 (17)	0.0297 (17)	0.0000 (13)	−0.0015 (13)	0.0027 (13)
C8	0.0353 (19)	0.0274 (17)	0.049 (2)	−0.0038 (15)	0.0000 (16)	−0.0047 (15)
C9	0.0400 (18)	0.0252 (15)	0.051 (2)	0.0026 (15)	0.0091 (17)	0.0056 (17)
C10	0.0346 (17)	0.0371 (18)	0.0344 (19)	0.0056 (14)	0.0054 (14)	0.0184 (14)
C11	0.0266 (16)	0.0325 (17)	0.0264 (18)	0.0005 (13)	0.0038 (12)	0.0050 (13)
Cl1	0.0487 (5)	0.0513 (5)	0.0262 (4)	−0.0041 (4)	−0.0028 (4)	−0.0125 (4)

### Geometric parameters (Å, º)

**Table:**

Zn1—N1	2.097 (2)	C4—H4	0.9500
Zn1—N1^i^	2.097 (2)	C5—C6	1.484 (4)
Zn1—Cl1^i^	2.2432 (11)	C6—C7	1.386 (4)
Zn1—Cl1	2.2432 (11)	C6—C11	1.392 (4)
N1—C1	1.347 (4)	C7—C8	1.384 (4)
N1—C5	1.362 (3)	C7—H7	0.9500
C1—C2	1.379 (4)	C8—C9	1.382 (5)
C1—H1	0.9500	C8—H8	0.9500
C2—C3	1.374 (4)	C9—C10	1.384 (5)
C2—H2	0.9500	C9—H9	0.9500
C3—C4	1.388 (4)	C10—C11	1.384 (4)
C3—H3	0.9500	C10—H10	0.9500
C4—C5	1.378 (4)	C11—H11	0.9500
			
N1—Zn1—N1^i^	96.30 (13)	N1—C5—C4	121.0 (2)
N1—Zn1—Cl1^i^	106.95 (7)	N1—C5—C6	118.6 (2)
N1^i^—Zn1—Cl1^i^	121.04 (6)	C4—C5—C6	120.4 (2)
N1—Zn1—Cl1	121.04 (6)	C7—C6—C11	119.6 (3)
N1^i^—Zn1—Cl1	106.95 (7)	C7—C6—C5	120.8 (2)
Cl1^i^—Zn1—Cl1	105.55 (7)	C11—C6—C5	119.6 (3)
C1—N1—C5	118.1 (2)	C8—C7—C6	120.7 (3)
C1—N1—Zn1	114.56 (19)	C8—C7—H7	119.6
C5—N1—Zn1	125.39 (18)	C6—C7—H7	119.6
N1—C1—C2	123.2 (3)	C9—C8—C7	119.3 (3)
N1—C1—H1	118.4	C9—C8—H8	120.3
C2—C1—H1	118.4	C7—C8—H8	120.3
C3—C2—C1	118.7 (3)	C8—C9—C10	120.5 (3)
C3—C2—H2	120.6	C8—C9—H9	119.7
C1—C2—H2	120.6	C10—C9—H9	119.7
C2—C3—C4	118.8 (3)	C9—C10—C11	120.1 (3)
C2—C3—H3	120.6	C9—C10—H10	120.0
C4—C3—H3	120.6	C11—C10—H10	120.0
C5—C4—C3	120.2 (3)	C10—C11—C6	119.8 (3)
C5—C4—H4	119.9	C10—C11—H11	120.1
C3—C4—H4	119.9	C6—C11—H11	120.1

Symmetry code: (i) −*x*, −*y*, *z*.

### Hydrogen-bond geometry (Å, º)

**Table:**

*D*—H···*A*	*D*—H	H···*A*	*D*···*A*	*D*—H···*A*
C11—H11···Cl1^ii^	0.95	2.90	3.666 (3)	138

Symmetry code: (ii) −*x*, *y*, *z*−1/2.

## References

[bb1] Bruker (2000). *SMART*, *SAINT* and *SADABS* Bruker AXS Inc., Madison, Wisconsin, USA.

[bb2] Flack, H. D. (1983). *Acta Cryst.* A**39**, 876–881.

[bb3] Samha, H. A., Martinez, T. J. & Armond, M. K. D. (1993). *Inorg. Chem.* **32**, 2583–2586.

[bb4] Santoro, A., Prokhorov, A. M., Kozhevnikov, V. N., Whitwood, A. C., Donnio, B., Williams, J. A. G. & Bruce, D. W. (2011). *J. Am. Chem. Soc.* **133**, 5248–5251. 10.1021/ja201245s 21413706

[bb5] Sheldrick, G. M. (2008). *Acta Cryst.* A**64**, 112–122. 10.1107/S0108767307043930 18156677

[bb6] Yoshinari, N., Kitani, N. & Konno, T. (2010). *Acta Cryst.* E**66**, m1499. 10.1107/S160053681004393X PMC3009048 21588912

[bb7] Zhao, Q., Lei Li, L., Li, F., Yu, M., Liu, Z., Yi, T. & Huang, C. (2008). *Chem. Commun.* pp. 685–687. 10.1039/b712416c 18478690

